# Power Density Titration of Reversible Photoisomerization of a Fluorescent Protein Chromophore in the Presence of Thermally Driven Barrier Crossing Shown by Quantitative Millisecond Serial Synchrotron X-ray Crystallography

**DOI:** 10.1021/jacs.3c12883

**Published:** 2024-06-07

**Authors:** James M. Baxter, Christopher D. M. Hutchison, Alisia Fadini, Karim Maghlaoui, Violeta Cordon-Preciado, R. Marc L. Morgan, Michael Agthe, Sam Horrell, Friedjof Tellkamp, Pedram Mehrabi, Yannik Pfeifer, Henrike M. Müller-Werkmeister, David von Stetten, Arwen R. Pearson, Jasper J. van Thor

**Affiliations:** † Department of Life Sciences, Imperial College London, London SW7 2AZ, U.K.; ‡ Center for Structural Biology, Imperial College London, London SW7 2AZ, U.K.; § Department of Physics, Center for Free-Electron Laser Science, Institute for Nanostructure and Solid State Physics, University of Hamburg, Hamburg 22607, Germany; ∥ Scientific Support Unit Machine Physics, Max-Planck-Institute for Structure and Dynamics of Matter, Hamburg 22761, Germany; ⊥ Max Planck Institute for the Structure and Dynamics of Matter, CFEL, Hamburg 22607, Germany; # Institute of Chemistry—Physical Chemistry, University of Potsdam, Potsdam 14469, Germany; ∇ European Molecular Biology Laboratory (EMBL), Hamburg 22607, Germany; ○ Institute for Nanostructure and Solid State Physics & The Hamburg Centre for Ultrafast Imaging, HARBOR, Universität Hamburg, Hamburg 22607, Germany

## Abstract

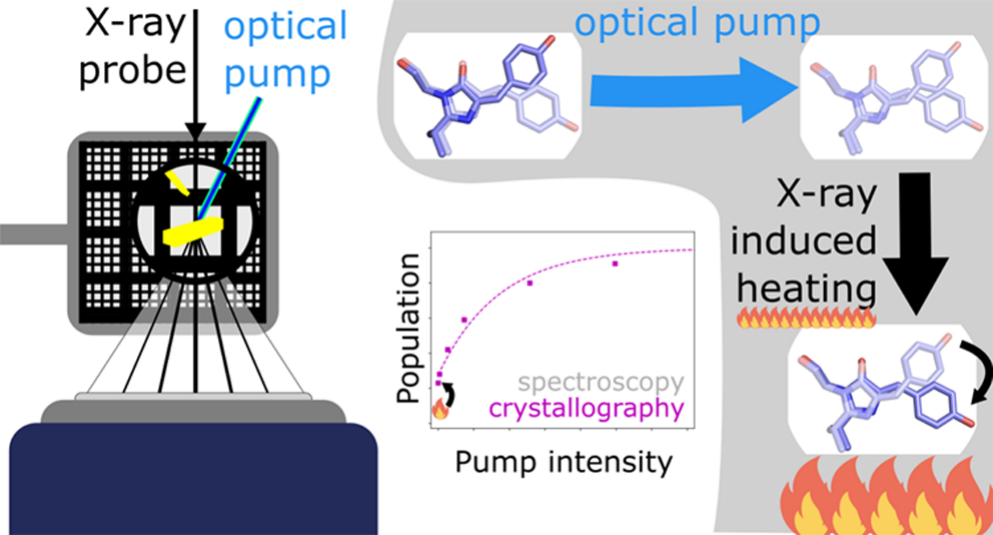

We present millisecond quantitative serial X-ray crystallography at 1.7 Å resolution demonstrating precise optical control of reversible population transfer from Trans–Cis and Cis–Trans photoisomerization of a reversibly switchable fluorescent protein, rsKiiro. Quantitative results from the analysis of electron density differences, extrapolated structure factors, and occupancy refinements are shown to correspond to optical measurements of photoinduced population transfer and have sensitivity to a few percent in concentration differences. Millisecond time-resolved concentration differences are precisely and reversibly controlled through intense continuous wave laser illuminations at 405 and 473 nm for the Trans-to-Cis and Cis-to-Trans reactions, respectively, while the X-ray crystallographic measurement and laser illumination of the metastable Trans chromophore conformation causes partial thermally driven reconversion across a 91.5 kJ/mol thermal barrier from which a temperature jump between 112 and 128 K is extracted.

## Introduction

The increasing brilliance of X-ray free electron lasers (XFELs) and synchrotrons has led to the development of serial crystallography, allowing for the collection of room temperature, time-resolved, atomic-resolution structures from protein crystals. Unlike conventional synchrotron and home-source-based single-crystal macromolecular crystallography, serial crystallography involves the collection of diffraction intensities from numerous crystals which are then merged to create a full data set.^1^ Serial femtosecond crystallography (SFX) at XFELs uses short (10–100 fs) and intense^2^ (1 mJ) hard X-ray pulses to obtain thousands of stationary diffraction patterns for measurement of time dependent structural movements on millisecond to subpicosecond time scales.^3−6^ SFX has driven significant progress in the data processing of stationary crystal diffraction patterns^7,8^ and the development of high-throughput crystal delivery mechanisms.^9,10^ More recently, these advances have been utilized at synchrotrons to enable serial synchrotron crystallography (SSX) for the collection of protein structures without the need for cryo-cooling.^11^ This allows for much shorter X-ray exposure times which significantly reduces the radiation dose per crystal^12,13^ and allows for time-resolved structural measurements^14−20^ using time-resolved SSX (TR-SSX).

In TR-SSX, reactions can be initiated using an optical laser “pump” pulse followed by a hard X-ray probe. Due to available X-ray flux and detector exposure times, TR-SSX measurements are not in the diffract-before-destruction regime,^1^ utilized in SFX, and therefore there remain open questions on the effects of the, relative to XFELs, long intense X-ray exposures used. Typically, a protein crystal will absorb around 2% of incident X-ray photons (∼12.4 keV) primarily through the photoelectric effect (1.5%) and Compton scattering (0.15%).^21^ This absorbed energy leads to stronger thermal vibrations as well as chemical reactions caused by the generation of radical species which can propagate considerable distances through the crystal lattice at higher temperatures.^22,23^ Of particular concern to TR-SSX studies is that increasing temperatures, as a result of the longer exposure times required as compared to XFEL studies, will exponentially decrease the lifetimes of intermediate states following Arrhenius behavior. Depending on the barrier size, even small increases in temperature can accelerate reaction rates beyond the time resolution of TR-SSX. For example, in the photoactive yellow protein (PYP) photocycle the PR2 to PB1 reaction must overcome a Gibbs Free Energy activation barrier of 51 kJ/mol.^24^ An increase of just 21 K from room temperature would decrease the lifetime of the PR2 state by around an order of magnitude.^24^

X-ray-induced heating has long been considered as a potential complicating factor in conventional macromolecular crystallography (MX).^23,25−28^ Adiabatic heating calculations, with millisecond X-ray exposure times and typical synchrotron microfocus monochromatic beamline X-ray fluxes (10^12^ photons/s), suggest heating of 50–70 K during data collection.^25^ Recently, Warren et al.^29^ directly characterized this heating effect using ruby microcrystals mounted in a loop, cooled to cryogenic temperatures. By monitoring the shift in the ruby fluorescence wavelength, they inferred X-ray induced temperature increases of only around 20 K, much lower than the calculated adiabatic increase, but consistent with a “KKT” model^29^ where heat is rapidly transferred to the surrounding environment, in this case, the 100 K dry nitrogen cryostream. However, in (TR-)SSX, there is currently no experimental characterization or quantification of direct sample heating using high-throughput serial crystallographic delivery methods. As the increasing brilliance of synchrotron will lead to shorter X-ray exposures, heating could become a major problem in TR-SSX, especially once X-ray exposure times become significantly shorter than thermal diffusion rates (on the order of μs for small (∼10 μm) crystals^28^). This highlights the need for improved understanding of X-ray-induced heating in TR-SSX measurements, to allow full advantage to be taken of the increased brilliance of low emittance synchrotron sources.^30,31^

This work demonstrates the use of SSX to extract accurate photoinduced structure factor amplitude differences on millisecond time scales from partial reflections, and recovery of the associated state occupancies. This analysis is supported by accurate control, using light titration, of photoproduct formation when SSX is directly compared with flash photolysis data. We also quantify the magnitude of X-ray and pump laser-induced heating that occurred while collecting a high-resolution TR-SSX data set using a fixed target delivery system. The system studied was a reversibly switchable fluorescent protein (rsFP) which is a representative of a class of proteins that undergo a reversible photolysis reaction from a highly fluorescent “on” state to a weakly fluorescence “off” state. The specific rsFP used, rsKiiro,^32^ is a mutant of Skylan-NS^33^ specifically developed for ultrafast time-resolved crystallographic studies due to its ability to form crystals that diffract to high resolution (∼1.1 Å for single-crystal cryo-MX), high photoswitching quantum yield (∼18% Trans-to-Cis in the crystal), and high protein expression yields. Structurally, rsKiiro is similar to green fluorescent protein (GFP) with an 11-stranded β-barrel surrounding an α helix. The chromophore of rsKiiro is formed from the Ala-Tyr-Gly tripeptide at residue numbers 62–64 in the α helix. During folding, an autocatalytic condensation reaction produces the 4-(*p*-hydroxybenzylidene)-5-imidazolinone (*p*-HBI) chromophore.^34,35^ At thermal equilibrium, the on phenolate-Cis chromophore state is dominant.^36,37^ Excitation at 488 nm drives the conversion from a Cis to a Trans-phenol state at pH 8, with a sequential deprotonation responsible for the high spectroscopic contrast between the two states.^38^ The chromophore isomerization is accompanied by a rearrangement of His194, Arg66, and Ser142 amino acids that surround the chromophore. In this study, we probed the light-induced reverse reaction, from the Trans to Cis chromophore conformation, initiated with 2 ms 405 nm pulses at a range of optical flash energy densities (0.1–6.7 mJ/mm^2^) after the initial Cis-to-Trans photoconversion had been driven to completion using ∼13 mW/mm^2^ of 473 nm CW illumination. The resulting precisely prepared population concentrations were quantified carefully by X-ray crystallographic and spectroscopic analysis. The high correspondence between X-ray and optical quantification shows that TR-SSX can accurately extract small concentration differences. The crystallographic yields do show an increased Cis-state yield as compared to the spectroscopic flash photolysis studies, which suggests an appreciable temperature jump caused by both the laser illumination and the millisecond X-ray exposure is driving ground state thermal recovery.

## Experimental Methods

### Crystallization

For flash photolysis and serial synchrotron crystallography, rsKiiro was batch crystallized in 25% w/v poly(ethylene glycol) 3350 in 0.2 M lithium sulfate, 0.1 M Tris-HCl, pH 8.5, with a final protein concentration of 15 mg/mL, and seeded with 0.5% v/v microcrystal slurry (1 × 10^7^ crystals/mL). Incubation for at least 12 h at 20 °C produced microcrystals with average size of ∼2 × 2 × 15 μm and densities ∼5 × 10^7^crystals/mL.

### Flash Photolysis

Crystalline samples, with absorption between 0.5 and 1 OD (at 505 nm), were prepared by crushing a microcrystal slurry between two glass coverslips (Hampton Research HR3-254), as described elsewhere.^39^ Samples were preconverted to the Trans state, by illumination with the unfocused beam of a 100 mW 488 nm CW diode laser for 1 s. The Trans-to-Cis reaction was driven by flashes (2 ms TTL gated) from a 405 nm CW diode laser, focused to 40 × 29 μm (fwhm) spot. A series of neutral density filters (3 to 0.05 OD) were used to attenuate the pump and provide a range of energy densities between 0.07 and 56.8 mJ/mm^2^. A third CW diode laser, at 505 nm, acted as the probe source. The change in sample absorption was monitored using an amplified photodiode (Thorlabs APD440A) and digital oscilloscope (picoTechnology, PicoScope 4000). The power and focal spot size of the probe were carefully chosen to minimize actinic behavior, whist still allowing sufficient signal-to-noise on the photodiode and overlap with the pump. Before and after each flash photolysis experiment, each sample was fully cycled between the Cis and Trans states to determine the total photo switchable populations and irreversible bleach.

### Serial Crystallography Data Collection and Reduction

**Figure 1:**
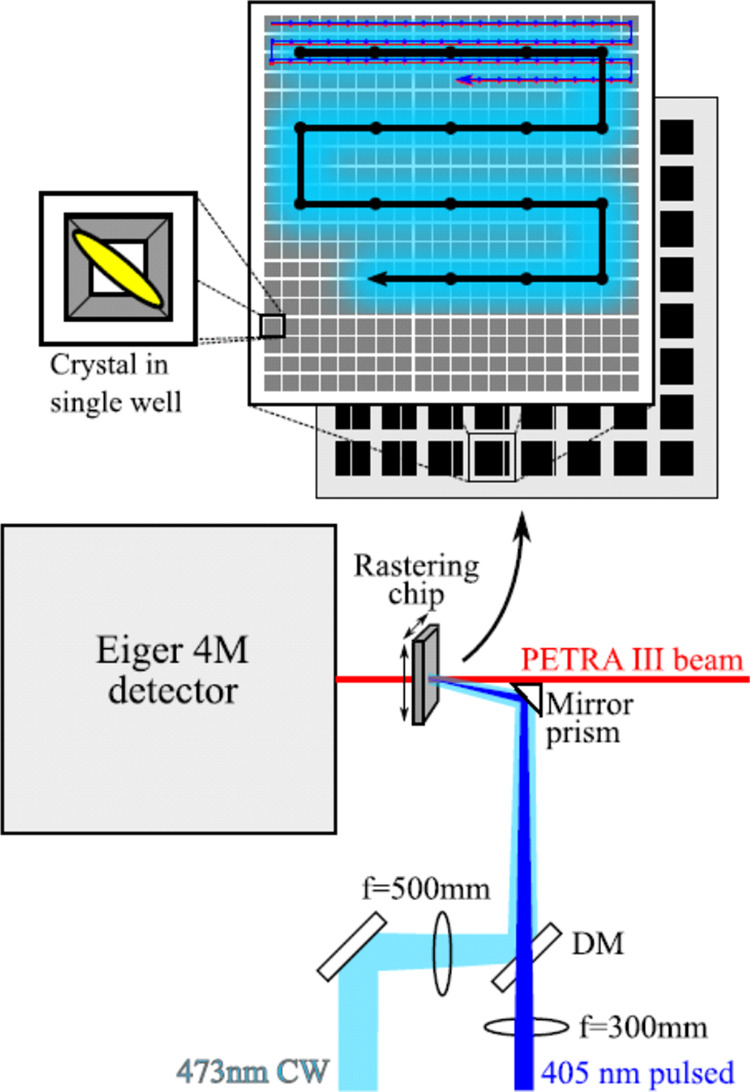
SSX fixed target data collection optical setup, showing the illumination pattern (Top) of the 473 nm preillumination which accumulates the Trans state (black arrow on light blue), the 405 nm pump (dark blue) which generates the Cis state, and X-ray beam (red) as well as the free space focusing geometry (bottom).

A fixed target sample delivery system using lithographed silicon chips, described elsewhere,^40,41^ was set up at the European Molecular Biology Laboratory (EMBL) end station P14.EH2 (T-REXX) at the PETRA III storage ring (DESY, Hamburg). To prepare the protein in the Trans state, a 473 nm, 10 mW continuous wave (CW) laser was loosely focused to around 1 mm (fwhm) on the chip and attenuated to 0.3W/cm^2^ using an ND filter (SI Section S3). The Trans–Cis photoreaction was driven by a 2 ms flash from a 405 nm laser TTL triggered diode laser, which was focused using a 300 mm lens to 73 μm by 23 μm (fwhm) spot, as measured by a fitted^42^ knife-edge scan. During data collection, the 473 nm laser spot was scanned over a 20 × 20 block of crystal containing “wells” in a 5 × 5 pattern (Figure 1, well center–center distance 150 μm), pausing at each position for 1 s, to allow every crystal in the block to be efficiently preconverted. The X-ray beam was not shuttered during this process, however the 5 × 5 pattern and large 473 nm spot size allowed every well to be illuminated without the X-ray beam passing directly over a well during preillumination cycles. After this, the stage was returned to the start of the block and the X-ray beam targeted sequentially to each individual well. At each well position, the stage controller triggered both the 405 nm pump laser, to flash the well, and X-ray detector, to collect a diffraction image. The X-ray exposure was 10 ms with a photon-flux of 1.9 × 10^12^ photons/s, an X-ray beam size of 15 × 10 μm, and a wavelength of 0.9801 Å. Diffraction data were recorded using a DectrisEiger 4 M detector.

X-ray diffraction images were initially indexed using the XGANDALF^43^ algorithm and intensities merged using CrystFEL’s Monte Carlo method.^44^ Conversion of intensities to structure factors was done using PHENIX’s reflection file converter and massage option.^45^ Initial phases were obtained from the rsKiiro resting state structure (PDB 7QLI(32)). Coordinates were refined using REFMAC5,^46^ initially using rigid-body refinement for occupancy refinement. Anisotropic B-factors and all atom refinement was used for the final structures deposited to the PDB with the occupancies determined from the minimization of difference density method introduced in the text below. Structure factors were brought onto an absolute scale using anisotropic scaling in SCALEIT within the CCP4i suite.^47^ Difference electron density maps were calculated from weighted structure amplitudes based on the Bayesian statistics analysis by Ursby et al.^48,49^ A weighting was applied to each structure factor and the associated error was propagated as implemented in previous works.^6,50,51^ Negative electron density integration above 2 rmsdat, a distance of 5 Å, from the chromophore was done using published Fortran code.^6^ Model building was done using COOT.^52^

### Occupancy Determination by Crystallographic Analysis

To analyze the occupancy of the different conformational states of the protein, three methods of crystallographic analysis were used: minimization of R-factors, minimization of difference electron density, and regeneration of the end state through extrapolated structure factor addition.^6,50^ Difference electron density maximum peaks were obtained using Coot.^52^

The R-factor minimization method involved varying the occupancy of a coordinate set from a full Cis occupancy to a full Trans. The models of the pure-Cis and pure-Trans structures were initially generated from previously reported cryo-MX structures^32^ (Cis: PDB 7QLI & Trans: PDB 7QLJ). These “pure” models were first refined against the 0 mJ/mm^2^(pure-Cis) and 14.43 mJ/mm^2^ (pure-Trans) data to account for changes in hydration and unit-cell size between the cryo-MX and room temperature data. Multiple conformations were removed from each model and the two coordinate sets combined into a single model using PHENIX.^45^ The occupancy was altered for the Cis and Trans states from 0% Cis (100% Trans) to 100% Trans (0% Cis) at 1% intervals. Each generated coordinate set was refined using 5 cycles of rigid-body refinement to find the occupancy ratio which minimized *R*_Work_ and *R*_Free_.

The second method involved minimization of the difference density around the chromophore. Weighted difference electron density maps of observed structure factors (*mF*_obs_) minus calculated structure factors (*nF*_calcd_) maps were generated for each 1% occupancy step. The occupancy ratio with the lowest integrated negative electron density in the chromophore region was used to determine the occupancy assignment and, therefore, population transfer (PT) from the initial Trans to the Cis state. Third, extrapolated maps were calculated using the Bayesian weighted difference density (calculated above) according to Ursby et al.^48,49^ Dark state (Cis) coordinates were used to calculate the structure factors and phases. Data sets were scaled, using the anisotropic option in SCALEIT, by resolution bin on a normalized absolute scale against the dark reference structure factors. The difference structure factors Δ*FQ* were added in a linear combination in multiples of *N*_EXT_ to the calculated Cis structure factors (*F*_Cis_) to generate extrapolated structure factors, *F*_EXT_ = *F*_Cis_ + *N*_EXT_ Δ*FQ*. The calculated structure factors from the refined Cis-only geometry fitted to the highest energy density illumination (and therefore highest Cis conformation occupancy) crystallography data were used for *F*_Cis_ to prevent any residual unconverted Trans conformer from skewing the analysis. The negative electron density around the chromophore was then integrated as a function of the *N*_EXT_. A buildup of negative electron density indicates the generation of a physical real-space structure and therefore an over inflated value of *N*_EXT_. A characteristic *N*_EXT_ was determined from the change in slope of the integrated electron density and related to the population transfer (PT) as PT = 200/*N*_EXT_.^6^ The change in the slope was determined by fitting two different linear regions. These regions were identified by finding the maximum value of the convolution of a step function with the zero-mean gradient, calculated as the difference between the gradient of the integrated DED and its mean. Two lines were iteratively fitted to these regions, with at least three successive values of *N*_EXT_ selected in each iteration, to find the best overall fit for two lines. The value of *N*_EXT_ was extracted at the intersection of the two lines.

## Results and Discussion

Flash photolysis measurements on crystals show that the Trans-state formation plateaus at 100% conversion for energy densities above 10 mJ/mm^2^, as seen in Figure S4. The single flash irreversible bleaching yield was found to be lower than 0.1% per flash at the maximum energy density used, allowing multiple measurements to be made on a single sample position. The average optical density of the samples was 0.95 at 505 nm. The data were modeled as described in the Supporting Information, similarly to Lin et al.^53^ and Bolton et al.^54^ The reaction followed first-order rate laws with no indication of biexponential behavior. A fluence-based first-order rate constant, k, of 0.274 mm^2^/mJ was fitted for the Trans to Cis reaction (off to on reaction).

A total of 435,200 images were obtained across 6 laser energy densities at 405 nm of 0, 0.1, 0.53, 1.78, 6.74, and 14.43 mJ/mm^2^. From these images, 79,976 single-crystal indexed diffraction patterns were obtained and merged into 6 data sets for each corresponding laser energy density. Initial comparisons of the serial crystallography data to cryo-MX data showed a decrease in resolution and an increase of 5% in unit-cell volume. As seen in Figure S5, the initial examination of the chromophore-omitted maps clearly shows increasing occupancy of the Cis state at higher energy densities. In the preilluminated (unflashed, 0 mJ/mm^2^) data, signals of the residual Cis population can be seen. This is due to thermally driven ground state isomerization occurring within the 10 ms duration of the X-ray probe due to laser and X-ray induced heating (discussed in detail below).

As seen in Table S2, data recorded at all laser energy densities showed good data reduction statistics and had similar signal-to-noise ratios and comparable Wilson B-factors. Data below a value of 0.5 in Fourier shell correlation^55^ were discarded, leading to a uniform limit of 1.7 Å being applied to all data sets. Refinement gave excellent model-electron density agreement with R-factors appropriate for this resolution.

**Figure 2:**
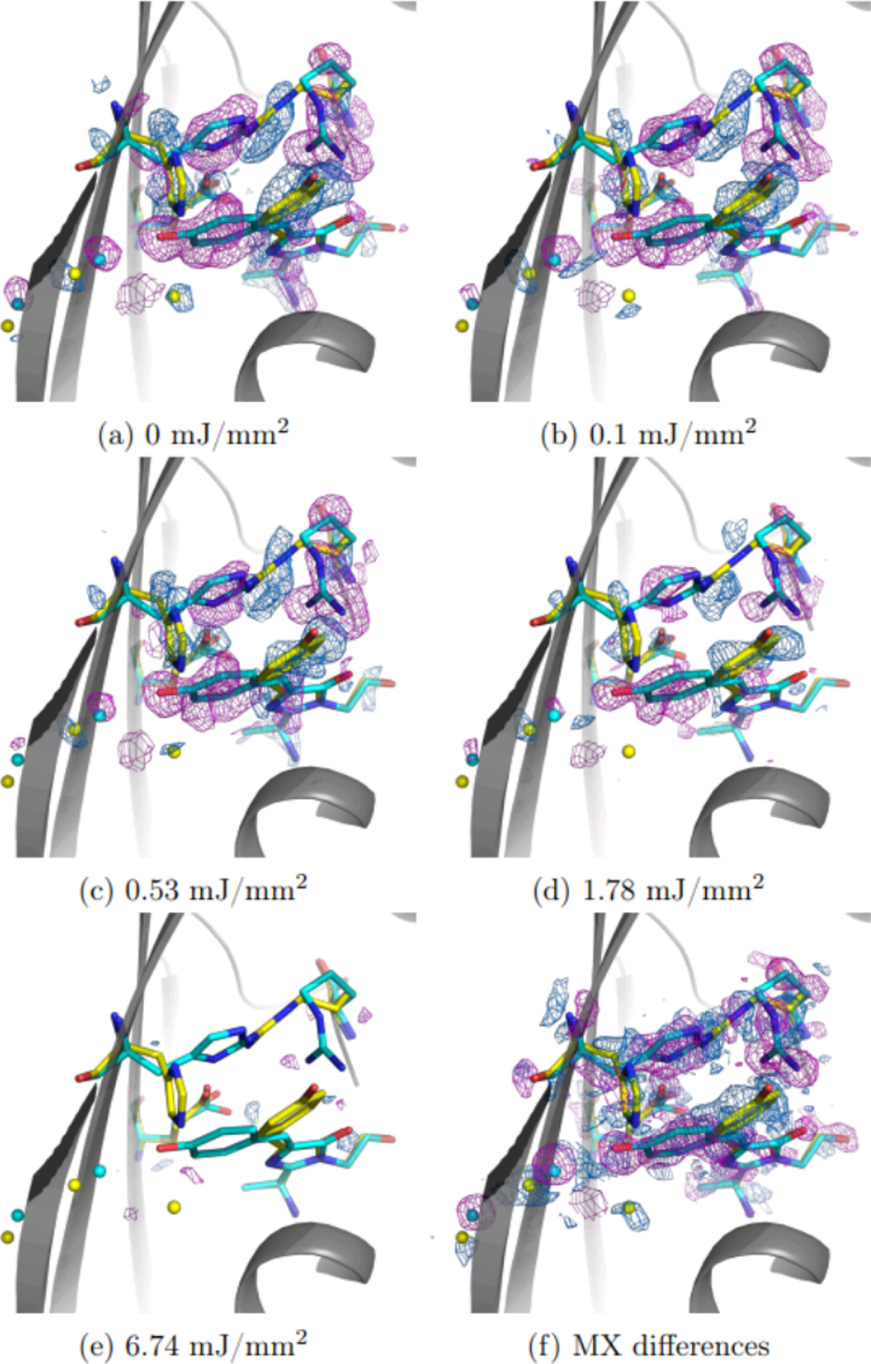
Power density titration of Trans-to-Cis photoisomerization using 2 ms flashes at 405 nm, following 473 nm continuous wave accumulation of the Trans state. Q-weighted difference electron density relative to the 14.43 mJ/mm^2^ illumination condition with dominating Cis state is plotted at ±3σ rms (positive in blue, negative is magenta), scaled by resolution bins. Cis coordinates are shown in cyan and Trans in yellow. Panels (a)–(e) show differences from the highest energy density (*E* = 14.43 mJ/mm^2^) structure factors: *F*_obs_(*E* = 14.43) – *F*_obs_(*E*). For comparison, panel (f) shows the difference of two steady state structures collected using MX reported previously.^32^

Examination of Q-weighted *F*_Obs_ (*E* = 14.43 mJ/mm^2^) – *F*_Obs_ (*E*) electron density difference maps (Figure 2a–e) reveals a strong change in reactant concentrations with signals overlapping the equivalent cryo trapped difference density (Figure 2f). In all cases, the strongest differences are seen on the chromophore phenol ring and residues Arg66, Water59, and His194. More subtle changes previously observed at 100 K along the protein backbone^32^ were also reflected in both refined coordinates and difference maps peaks. This agreement of TR-SSX and cryo-MX signals suggests that the isomerization and rearrangement of the chromophore environment are fully completed within the 10 ms X-ray exposure time which implies rsKiiro undergoes similar photochemistry to other proteins in its class, such as Dronpa, the most widely studied rsFP.^36,56^

Absorbed dose calculations using RADDOSE3D^57,58^ estimate a dosage of 0.062 MGy per crystal which is below the estimated maximum room temperature tolerable dose limit of 0.38 MGy.^12^ Furthermore, the phased electron density shows no signs of specific radiation damage, and merging statistics such as the Wilson B factor is acceptable. This means the decrease in resolution 1.16–1.7 Å (cryo-MX to TR-SSX) is most likely due to decreased crystal size and hence diffracting power, rather than radiation damage effects.

R-factor analysis, as seen in Figure S8, shows minima of 0.20 and 0.23 for *R*_Work_ and *R*_Free_, respectively, for all energy densities. The assigned population transfer follows the same general trend as the flash photolysis data but is offset by around 20%. There is also a discrepancy between the *R*_Work_ and *R*_Free_ population assignments. This is most likely due to the smaller number (5%) of *R*_Free_ reflections being insensitive to small changes in the structure.

**Figure 3:**
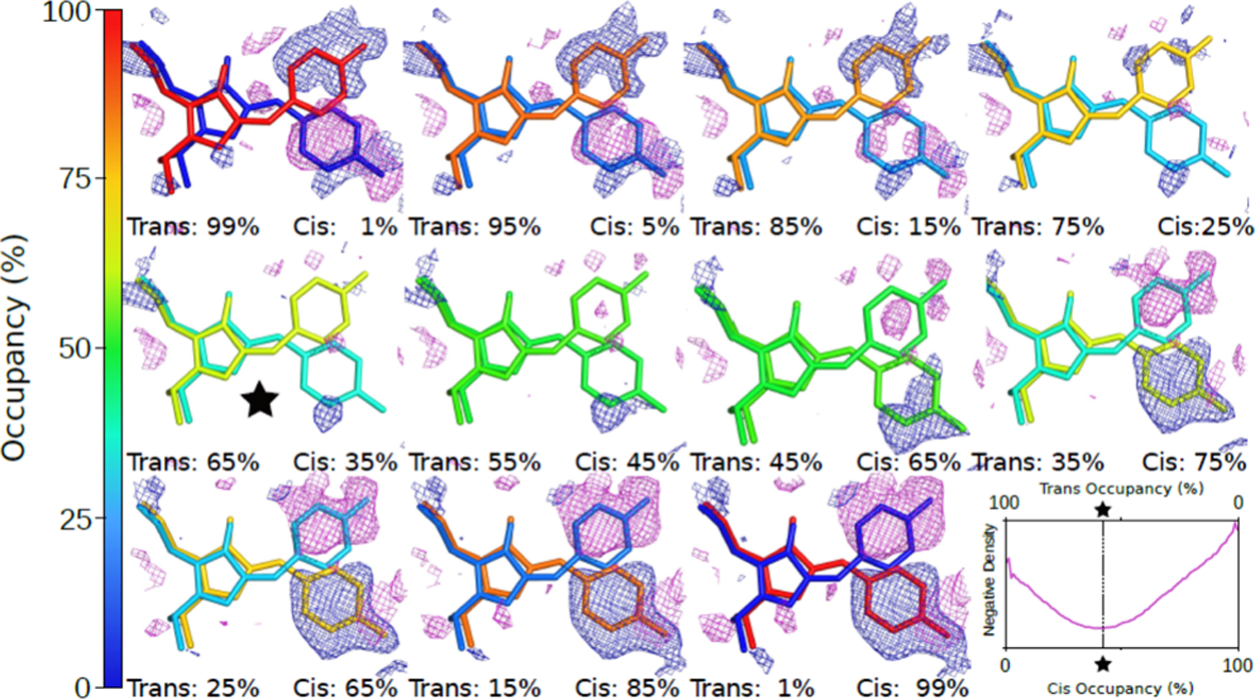
Difference electron density for 0.53 mJ/mm^2^ energy density: *mF*_obs_ (0.53) – *nF*_calcd_ (Occupancy). Plotted at ±2.0 rms with negative and positive features in magenta and blue, respectively. The percentage indicates the cis occupancy fraction. In the bottom right, the integrated negative electron density around the chromophore is plotted as a function of the Cis occupancy with the minimum indicated.

**Figure 4:**
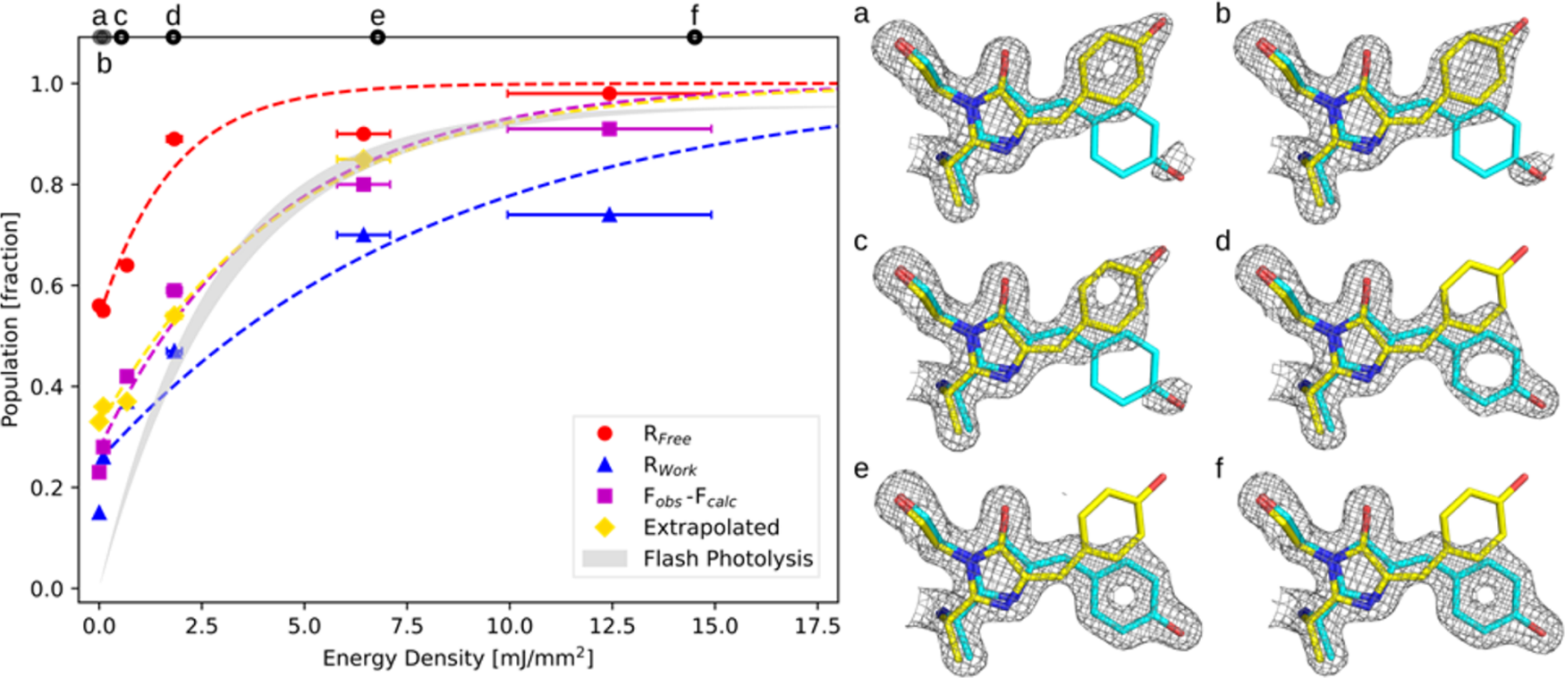
Left: Population fraction of the Cis state is plotted as a function of energy density state for the different occupancy determination methods. The fluence-based first-order rate constants, *k*(mm^2^/mJ) and retrieved temperature jump values are reported for the four different crystallographic quantification methods. Right: (a) (0 mJ/mm^2^), (b) (0.10 mJ/mm^2^), (c) (0.53 mJ/mm^2^), (d) (1.78 mJ/mm^2^), (e) (6.74 mJ/mm^2^), (f) (14.43 mJ/mm^2^) phased electron density maps at 1.5 rms using occupancies determined by minimization of 2*F*_obs_ – *F*_calcd_ density with increasing energy density of the 405 nm 2 ms flashes. A clear shift in electron density can be seen toward the photoproduct (Cis state) as the energy density increases. The crystallographic determinations of Cis population at zero energy density were 0.56 ± 0.05 (*R*_Free_ method), 0.33 ± 0.05 (*F*_extrapolated_), 0.25 ± 0.05 (*R*_Work_), and 0.29 ± 0.05 (*F*_o_ – *F*_c_).

Minimization of the *F*_obs_ – *F*_calcd_ density, illustrated in Figure 3, shows clearly that higher flash energies give larger population transfers (Figure 4). A clear difference in reaction yield can be seen even when comparing the lowest flash energies (0.00 and 0.10 mJ/mm^2^), where a difference of 3% population transfer is determined from the shift in the minima. This method of population determination was also found to have a lower dependency on supplied model coordinates compared to R-factor minimization or the extrapolated determination. This is most likely due to the site-specificity of integrating only around the chromophore region. It was found that perturbed starting coordinates with intentionally introduced coordinate errors still gave population transfers within 10% as long as the errors were not in the chromophore region.

*Q*-weighted difference maps, seen in Figure 2, show the progression of the reaction as differences around the chromophore become less pronounced with the larger flash energy densities. Extrapolated structure factors were calculated using the highest flash energy (14.43 mJ/mm^2^) state as the phase source. This was to account for any residual Cis state which may have not been fully preconverted. The characteristic *N*_EXT_ values follow the trend of increasing Cis occupancy with energy density but were found to be extremely sensitive to starting phases, calculated model structure factors, and data reduction protocol.

As can be seen in Figure 4, the minimization of weighted difference density follows the flash photolysis data most closely, with the derived rate constant differing by only 14%. The higher estimate of population and larger rate constant from the minimization of the *R*_Free_ method is likely due to the low number of reflections used in calculation of the *R*_Free_ metric. Occupancy analysis using *R*_Work_ follows a trend similar to that of the difference map minimization but is offset by approximately 30% and overall gives the lowest estimated photolysis rates. It is possible this is due to a form of model bias. If the Cis structures were not fully refined or had significantly different Debye–Waller factors compared to the Trans-state, the R-factor would be expected to shift in the Cis direction. To test this assumption, Cis-models with errors were refined to test the robustness of each occupancy refinement method. It was found that the difference map minimization still gave similar trends, while R-factor minimization and, especially, the extrapolated map method were very sensitive to changes in model coordinates. The extrapolated (Figure 4, yellow) map population determination yields a rate constant very similar to that of the minimization of difference densities (*F*_obs_ – *F*_calcd_, Figure 4 pink). Overall, this analysis suggests that a localized approach to occupancy refinement (i.e., difference density minimization) is effective at extracting population information from our data.

Four different crystallographic methods have quantified the percentage population of residual Cis state measured under laser illumination at 473 nm wavelength (Figure 4). We conclude that the initial population offset seen in the crystallographic data at 0 mJ/mm^2^ energy density of the 405 nm illumination is due to increased thermal recovery caused by X-ray induced heating. Based on the spectroscopic determination of the laser-driven Cis-to-Trans conversion rate, the measured optical power density, and profile, in addition to a small contribution of thermal back-conversion from Trans to Cis prior to X-ray exposure, an initial offset in population fraction from optical measurements is estimated to be 12.5% (SI Section S3). The crystallographic determination of Cis concentration at zero power density of the 405 nm laser is systematically larger than the spectroscopy-based determination (Figure 4 and Tables S1–S3). The difference between the crystallographic and spectroscopic quantifications is assigned to the *x*-induced heating, which drives population across a 91.5 kJ/mol barrier to reform the Cis state (Figure 4).

Calculations suggest that laser-induced heating can be neglected for the data analysis in Figure 4, although some contribution is expected for the higher power density conditions. This is also confirmed by the observation that flash photolysis measurements do not show residual Cis state under 488 nm illumination (Supporting Information, Section S4.2). The thermal activation barrier of the Trans-to-Cis reaction has been previously reported to be 91.5 ± 5 kJ/mol with a rate *k*_1_ = 4.08 × 10^–4^ s^–1^ at 293 K.^32^ As described in the Supporting Information (SI Section S4.3), this activation energy barrier was used to estimate the magnitude of X-ray induced heating (Table 1).

**Table 1:** Full Results of X-ray Crystallographic Temperature Modeling Using the Occupancies Derived with the Four Methods of Occupancy Refinement^a^

method	*k* (mm^2^/mJ)	Δ*T* (K)	δ*T* (K)
*R*_Free_	0.562	128.8	111.3–152.9
*R*_Work_	0.121	112.1	97.4–132.0
*F*_obs_ – *F*_calcd_	0.235	115.9	100.6–136.8
extrapolated	0.218	118.9	103.0–140.5
flash photolysis	0.274	N/A	N/A

a Described in the Experimental Methods section. The temperature increase is denoted as Δ*T* and 95% confidence intervals, δ*T* calculated from error of *E*_a_ and [Cis] at 0 mJ/mm^2^ (Figure 4). The fluence-based first-order rate constant, *k*, is found from fit of the data in Figure 4 and is defined in Section S4.2.

Temperature increases due to X-ray heating from 112.1 to 128.8 K were extracted (97.4–152.9 K at 95% confidence interval, Table 1). It is found that the uncertainty of the *E*_a_ value dominates the uncertainty of the temperature jump value that is calculated (SI Sections S3, S4.3, Tables S1–S3).

Calculation of the temperature jump based on the RADDOSE calculation of the absorbed dose strongly depends on the heat capacity and modeling of the cooling (SI Section S1). In the absence of cooling within the 10 ms probe window, the temperature jump is found from
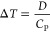
where *D* = 0.062 MGy is the calculated absorbed Dose and *C*_p_ is the heat capacity (J K^–1^ kg^–1^). At 300 K temperature for pure protein *C*_P_ = 5 × 10^2^ J K^–1^ kg^–1^, which would give rise to Δ*T* = 119 K. The measured *C*_P_ values for lysozyme crystals, taken as a reference, very strongly depend on the hydration level, as shown by Miyazaki et al.^59^ At 300 K, for “wet” lysozyme crystals containing 45.7 wt % water, *C*_P_ was measured at 73.6 × 10^3^ J K^–1^ mol^–1^ = 4.91 × 10^3^ J K^–1^ kg^–1^. For this value of *C*_P_, Δ*T* = 13 K. At 300 K, for “dry” lysozyme crystals containing 7.4 wt % water, *C*_P_ was measured at 23.8 × 10^3^ J K^–1^ mol^–1^ = 1.59 × 10^3^ J K^–1^ kg^–1^. For this value of *C*_P_, Δ*T* = 39 K. The effect of cooling is estimated based on experimental measurement of heat-transfer coefficients of lysozyme crystals reported by Fujiwara^60^ (SI Section S1). The crystal heat capacity also strongly determines the characteristic time constant for the cooling as well as the volume of water surrounding the crystals (SI Figure S1). Assuming an infinite cooling bath, the maximum temperature jump calculated from the coupled rate equations is estimated to be between 30 and 80% of the temperature jump calculated from the absorbed Dose and *C*_p_. Under nearly dry conditions, Δ*T* can approach 100% of the value calculated from the ratio of *D*/*C*_p_. These calculations result in lower values for Δ*T* compared to the experimental measurements presented here, although there is overlap with the lower values in the 95% confidence range (Figure 4 and Table 1).

There are considerable uncertainties in the calculation strategies summarized and demonstrated (SI Section S1). First, these depend fully on the accuracy of the models used by RADDOSE3D^57,58^ to calculate the absorbed Dose as well as the accuracy of the physical parameters that are used for input. Warren et al. calculated temperature jumps for comparable X-ray absorption conditions in excess of 100 K in the absence of cooling, albeit at low temperature which decreases the heat capacity that is approximately proportional to absolute temperature.^29^ They concluded from calculation and measurement that cryogenic cooling under continuous flow results in characteristic cooling times in the range of 30 ms X-ray probe times used in that study, which result in significant reduction of the temperature jump as expected.

Under our experimental conditions, the magnitude of the temperature jump likely indicates a low value of the crystal heat capacity and poor cooling, as judged from the calculations. It is possible that, in addition to drying to air, the preconversion to the Trans state using the partially focused 10 mW 473 nm CW diode laser for 1 s resulted in the loss of water from both the crystal as well as the surrounding droplet.

The calculated values for the temperature jump depend on the accuracy of the activation energy for the thermal Cis generation, the steady state temperature experienced by the crystals prior to the X-ray exposure, and the accuracy of the laser power and profile for the preconversion. Section S4.3 demonstrates that the uncertainty for the activation energy of 91.5 ±5 kJ/mol strongly dominates the uncertainty of the calculated temperature jump.

These results illustrate the importance of controlling and characterizing the physical parameters that affect heating and cooling in time-resolved TR-SXX experiments that include the use of intense cw lasers as well as intense millisecond X-ray probing.^28,29,59,60^ Calculations suggest, however, that the temperature jump should be reduced under wet conditions and increased thermal cooling.

## Conclusions

In this study, we have used the sample itself as a thermometer to determine the temperature jump under conditions of TR-SSX from crystallographic information. Effectively, we exploit the knowledge of thermodynamic parameters for thermal conversion to determine the experimental temperature. We show that thermally driven ground state isomerization of a biological chromophore occurs as a result of millisecond X-ray exposure using the synchrotron serial crystallography method. This relies on quantitative crystallographic determination of small population differences being accurately extracted from crystallographic data measured using TR-SSX. The similarity of the TR-SFX data and equivalent spectroscopy measurement implies that TR-SSX experiments do not significantly perturb proteins during measurement when transitions involve large barriers, such as the ground state double-bond isomerization barrier (studied here). We show that the magnitude of X-ray induced heating is sufficiently high that it should be accounted for when studying systems with low activation energy barriers. Our experiments have shown that ground state double-bond isomerization across a 91 kJ/mol thermal barrier proceeds in the 10 ms exposure time used for the time-gated snapshot crystallography experiments. Because of the considerable magnitude of the barrier, the X-ray driven acceleration of the thermal rate is 3-4 orders of magnitude. This implies that lower barriers will be driven to completion under similar conditions. On the other hand, temperature jumps could serve to initiate reactions, with high-frame-rate detectors able to bin reaction evolution on millisecond time scales. The amplitude of difference electron density differences depends on the magnitude of the real-space displacement, the scattering cross sections, X-ray wavelength, space group symmetry and dimensions, the accuracy of provided phases, and Debye–Waller factors along with efficient and accurate reaction initiation. This is the first quantitative study showing a clear correlation between spectroscopy and serial crystallographic data derived from intermediate reactant concentrations.

Currently, many international synchrotron facilities are undergoing upgrades to deliver much lower emittance machines which will increase X-ray brightness by 1–2 orders of magnitude compared to current levels.^30,31^ Both the source brightness improvements and detector development will serve to improve the time resolution of TR-SSX experiments. This in turn will also improve the limitations of thermal barrier crossing and our ability to measure it in TR-SSX.

## Supporting Information Available

High-resolution SSX power titration chromophore-omit maps, occupancy fitting, crystallographic data tables, modeling of laser and X-ray heating, absorption spectra, flash photolysis error propagation and modeling, knife-edge fitting. Coordinates and structure factor amplitudes have been deposited in the PDB database. For preconverted (Trans) rsKiiro under different 400 nm illumination conditions: 0 mJ/mm^2^ (PDB 8UL0), 0.1 mJ/mm^2^ (PDB 8UL1), 0.53 mJ/mm^2^ (PDB 8UL2), 1.78 mJ/mm^2^ (PDB 8UL3), 6.74 mJ/mm^2^(PDB 8UL4), and 14.43 mJ/mm^2^ (PDB 8UL5) (PDF)
