# Supplementary material for: Power Density Titration of Reversible Photoisomerization of a Fluorescent Protein Chromophore in the Presence of Thermally Driven Barrier Crossing Shown by Quantitative Millisecond Serial Synchrotron X-ray Crystallography
Source: J Am Chem Soc. 2024 Jun 7;146(24):16394–403. doi: 10.1021/jacs.3c12883 (PMC11191680; doi:10.1021/jacs.3c12883)
Supplement: Supplementary file 1 — ja3c12883_si_001.pdf [file ja3c12883_si_001.pdf]

SUPPORTING INFORMATION

Power density titration of reversible Photoisomerization of a Fluorescent Protein chromophore in the presence of thermally driven barrier crossing shown by quantitative millisecond Serial synchrotron X-ray Crystallography

James M. Baxter,[1] Christopher D.M. Hutchison,[1] Alisia Fadini,[1] Karim Maghlaoui,[1] Violeta Cordon-Preciado,[1] R. Marc L. Morgan,[2] Michael Agthe,[3] Sam Horrell,[3] Friedjof Tellkamp,[4] Pedram Mehrabi,[5] Yannik Pfeifer,[6] Henrike M. Müller-Werkmeister,[6] David von Stetten,[7] Arwen R Pearson,[8] and Jasper J. van Thor[1,*]

[1]Department of Life Sciences, Molecular Biophysics, Imperial College London, London, UK.
[2]Center for Structural Biology, Imperial College London, London, UK.
[3]Department of Physics, Center for Free-Electron Laser Science, Institute for Nanostructure and Solid State Physics, University of Hamburg, Germany.
[4]Scientific Support Unit Machine Physics, Max-Planck-Institute for Structure and Dynamics of Matter, Hamburg, Germany.
[5]Max Planck Institute for the Struc-ture and Dynamics of Matter, CFEL, Hamburg, Germany.
[6]Institute of Chemistry - Physical Chemistry, University of Potsdam, Germany.
[7]European Molecular Biology Laboratory (EMBL), Hamburg, Germany.
[8]Institute for Nanostructure and Solid State Physics & The Hamburg Centre for Ultrafast Imaging, HARBOR, Universität Hamburg, Hamburg, Germany.
*Corresponding author. Email: j.vanthor@imperial.ac.uk

Table of Contents

S1. Adiabatic X-Ray Heating 2
S2. Laser Heating 2
S3. rsKiiro Spectra, Absorption Coefficient and Preconversion 3
S4. Photolysis Modelling 3
S4.1. Single Flash Yield and Error Propagation 3
S4.2. Flash Photolysis Model 5
S4.3. X-Ray Crystallography Photolysis Modelling 6
S5. Knife Edge Laser Focal Spot Size Determination 7
S6. SSX Energy Titration Chromophore-Omit Maps 8
S7. SSX Energy Titration Occupancy Determination by Minimisation of R_{Free} and R_{work} 9
S8. SSX Energy Titration Occupancy Determination by Extrapolated 9
S9. Crystallographic Data Tables 10
S10. Supporting Information References 10

S1. ESTIMATION OF ADIABATIC X-RAY INDUCED HEATING IN FIXED TARGET TR-SSX

Where *D* is the maximum dosage in Gy calculated using RADDOSE3D[1,2].

The input file used, using the values is

```
########################################################################################
#                         Crystal Block                         #
########################################################################################
Crystal
Type Cuboid
PixelsPerMicron 1
Dimensions 2 2 15
AbsCoefCalc RD3D
UNITCELL 39.6 74.5 78.9 90 90 90
NumMonomers 4
NumResidues 218
ProteinHeavyAtoms S 10
SolventHeavyConc S 200
SolventFraction 0.54


########################################################################################
#                          Beam Block                          #
########################################################################################

Beam
Type Gaussian
FWHM 30 10
Collimation Rectangular 15 10
FLUX 1.9e12
ENERGY 12.7


########################################################################################
#                          Wedge Block                          #
########################################################################################

Wedge 0 0.001
ExposureTime 0.01
ANGULARRESOLUTION 1
STARTOFFSET 0 0 0
TRANSLATEPERDEGREE 0 0 0
ROTAXBEAMOFFSET 0
```

The RADDOSE result:

Cell volume:
232770.78
Angstroms cubed.

Cuboid (Polyhedron) crystal of size [2, 2, 15] um [x, y, z] at a resolution of 1.00 microns per voxel edge.

Simple DDM.

Gaussian beam, 15.0x10.0 um with 30.00 by 10.00 FWHM (x by y) and 1.9e+12 photons per second at 12.70 keV.

Crystal coefficients calculated with RADDOSE-3D.
Photelectric Coefficient: 2.25e-04 /um.
Inelastic Coefficient: 2.00e-05 /um.
Elastic Coefficient: 1.93e-05 /um.
Attenuation Coefficient: 2.64e-04 /um.
Density: 1.20 g/ml.

Average Diffraction Weighted Dose : 0.030980 MGy
Last Diffraction Weighted Dose : 0.061650 MGy
Elastic Yield : 1.89e+05 photons
Diffraction Efficiency (Elastic Yield/DWD): 6.09e+06 photons/MGy
Average Dose (Whole Crystal) : 0.061948 MGy

Average Dose (Exposed Region) : 0.061948 MGy
Max Dose : 0.063020 MGy
Average Dose (95.0 % of total absorbed energy threshold (0.06 MGy)): 0.063054 MGy
Dose Contrast (Max/Threshold Av.) : 1.00
Used Volume : 100.0%
Absorbed Energy (this Wedge) : 4.57e-09 J.
Dose Inefficiency (Max Dose/mJ Absorbed) : 13787.5 1/g
Dose Inefficiency PE (Max Dose/mJ Deposited): 14087.5 1/g

The temperature change ΔT as a result of deposition of this absorbed dose in the absence of cooling depends on the heat capacity Cp

$$\Delta T = \frac{D}{C_P}$$

The heat capacity of rsKiiro crystals is unknown. Estimates can be taken from experimental measurements of pure protein[3] and for lysozyme crystalsreported for varying degrees of hydration [14]

At 300K temperature for pure protein $C_P = 5 \times 10^2$ J K^{-1} kg^{-1}, which would give rise to ΔT=124K. The measured C_P values for lysozyme crystals, taken as a reference, very strongly depend on the hydration level, as shown by Miyazaki *et al.*[5]. At 300K, for 'wet' lysozyme crystals containing 45.7wt% Water C_P was measured at 73.6×10^3 J K^{-1} mol^{-1}= 4.91×10^3 J K^{-1} kg^{-1}. For this value of C_P, ΔT=13K At 300K, for 'dry' lysozyme crystals containing 7.4wt% Water C_P was measured at 23.8×10^3 J K^{-1} mol^{-1} = 1.59×10^3 J K^{-1} kg^{-1}. For this value of C_P, ΔT=39K.

In the presence of cooling, an estimate of a characteristic cooling time has been described[4]. For small particles submerged in liquids the characteristic cooling time τ_c:

$$\tau_c \cong \frac{c_{ps}\rho_s L}{6h} \approx 0.3\frac{c_{ps}\rho_s L^{3/2}\nu^{1/2}}{\kappa u^{1/2}}$$

Where c_{ps} is the sample specific heat (J K^{-1} kg^{-1}), ρ_s the density (kg/m^3), L is the characteristic sample size (m), u is the velocity of the cooling medium, ν is the kinematic viscosity (10^{-6} m^2 s^{-1}) and κ_s is the thermal conductance (W m^{-1} K^{-1}) given by the Biot number,Bi:

$$Bi = \frac{hL}{\kappa_s}$$

Where h is the heat transport coefficient per unit area (J K^{-1} m^{-2} s^{-1}).

At 300K, Kriminski et al provide a value of κ_s=0.61 W m^{-1} K^{-1} for liquid water and $\nu=\eta/\rho$=1,002 x 10^{-6} m^2 s^{-1}.The heat transport per unit area h (J K^{-1} m^{-2} s^{-1})

$$h \cong \frac{\kappa}{\delta_t} = 0.6\frac{\kappa u^{1/2}}{(L\nu)^{1/2}}$$

where δt is the average thickness of the laminar thermal boundary layer (m)

In the absence of flow of the coolant, the heat transfer is best approximated from heat convection using Newton's heat transfer law. An overall heat-transfer coefficient U is defined for two media from the additivity of thermal resistances

$$\frac{1}{U} = \frac{1}{h_{crystal}} + \frac{1}{h_{water}}$$

Experimental measurement of thermal conductivity of lysozyme crystals were reported by Fujiwara *et al.*[6] with a value of 0.41 W m^{-1} K^{-1}. The effective heat-transfer coefficient U is calculated as U = 0.25 W m^{-1} K^{-1}.

An estimate of the characteristic cooling time is made using this value

$$\tau_c \cong \frac{c_{ps}\rho_s L}{U}$$

The calculated values for the characteristic cooling time again depend strongly on the heat capacity of the crystal. For pure protein (C_P = 5 x 10^2 J K^{-1} kg^{-1}) τ=4.8 ms, for 'wet' lysozyme crystals containing 45.7wt% Water(4.91 x 10^3 J K^{-1} kg^{-1}.) τ=47 ms and for 'dry' lysozyme crystals containing 7.4wt% Water (23.8 x 10^3 J K^{-1} kg^{-1}) τ=12 ms.

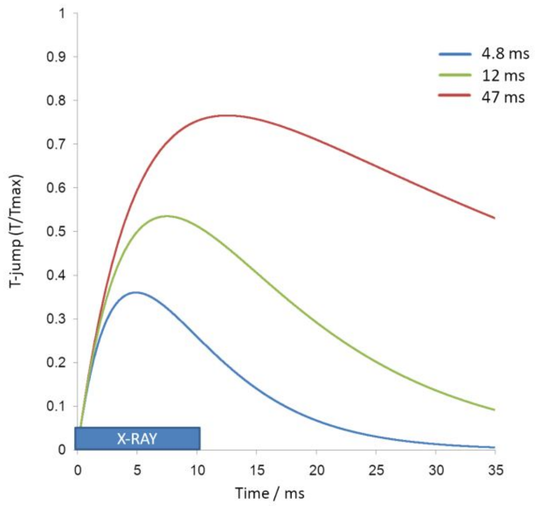

Fig S1. Illustration of temperature jump temporal profile assuming an effective heating time constant of τ= 5 ms and characteristic cooling times of 4.8 ms (blue), 12 ms (green) and 47 ms (red). The calculation assumes an infinite Volume for surrounding water using convection heat transfer. The temperature jump is plotted as a fraction of ΔTmax, which is the value in the absence of cooling ΔT=D/cp

The above example illustrates the calculation of potential heating profiles where crystals are embedded in stationary water reservoir with a characteristic dimension δ> 3L, in which case the final temperature is within less than 1 % of the reservoir initial temperature.
However, for less surrounding water, the maximal temperature jump will be higher if the final temperature is consequently increased.

S2. LASER HEATING

The measurements involved the use of two cw laser illuminations. The initial pre-conversion using the 473nm cw laser generates the metastable Trans state. Given that the characteristic cooling time is in the millisecond range, the rsKiiro crystals in the Trans state are assumed to be fully thermally equilibrated. The Power density titration series for the 405nm cw laser flash with 2ms duration, starts at a zero density. The crystallographic determination of remaining Cis state (Fig 2a, Fig 4, data point a) does not include the 405nm illumination and is used for the calculation of temperature jump caused by X-ray heating only (section S4.3). With subsequent increased power density up to 14.43 mJ/mm2 density and 2 ms duration, a saturating concentration of reversibly formed Cis state is measured (Figure 2, 4).
An estimate of the laser heating can be made. rsKiiro is not a static absorber, and photochromic bleaching of the electronic absorption band at 400nm proceeds with a 18% quantum yield[7]. Taking into account an additional 5% fluorescence quantum yield, the maximum absorbed dose (J/kg) can be estimated:

$$D_{405nm\text{ - }laser} = \frac{\mathrm{h}\nu\, N_A[rsKiiro](1 - QY_{fluo})}{QY_{switch}\rho_{cryst}}$$

Where N_A is Avogadros number (mol^{-1}), ρ_{cryst} is the density of the crystal (kg/l), the QY values for photoisomerization and fluorescence are given as fractions. [rsKiiro] in crystals is 48 mM

This estimate provides an upper value of the absorbed dose $_{D405nm\text{-}laser}$ = 6.31 x 10^4 J kg^{-1}.This values is directly comparable to the calculated absorbed x-ray dose.

The estimate of the optical laser induced heating follows the same considerations as the x-ray heating above in section S1. This value of 6.31 x 10^4 J kg^{-1} is however applied to condition labelled f in figure 4d. In the titration series, from 0 to 14.4 mJ/mm^2 the absorbed laser dose will follow the concentration profile of the product, hence will follow the exponential growth profile as shown. For instance, condition e (6.74 mJ/mm^2) shows between 40% and 60% population increase relative to condition a, hence an absorbed dose of about half the absorbed x-ray dose is estimated to contribute to the temperature jump in that condition. As a result, a small deviation from a first-order behaviour is expected, but cannot be resolved with the obtained accuracy of the Cis population presented in Figure 4.

S3. ESTIMATED RSKIIRO PRECONVERSION EFFICIENCY

To calculate the preconversion efficiency, the relative rate of switching in rsKiiro at lower power and optical densities was scaled to the laser and crystal optical density used in the TR-SSX experiment. rsKiiro absorbs most strongly at 485 nm with a calculated absorption coefficient of 98,727 $M^{-1}cm^{-1}$. At the flash wavelength of 405 nm the absorption coefficient is calculated as 31,184 $M^{-1}cm^{-1}$. Steady state measurements at a power density of 0.0315 W/cm^2 (at 489 nm) gave a switching rate of 0.32 s^{-1} in rsKiiro at 298 K and pH 8.4. The power density of the 473 nm laser on the chip is 0.30 W/cm^2 which scales the rate constant to 2.50 s^{-1} when accounting for change in absorption between 473 nm and 488 nm (a ratio of 0.82:1). Over the one second illumination period this should convert 92% of the protein to the trans state. The average waiting time for a crystal once preconverted was 45 s during which time an estimated 4.3% of the population will thermally recover back to the cis state on average. The thermal recovery rate constant at room temperature was previously reported as 1.0x10^{-3} s^{-1} [7] (Data in Fig S2)

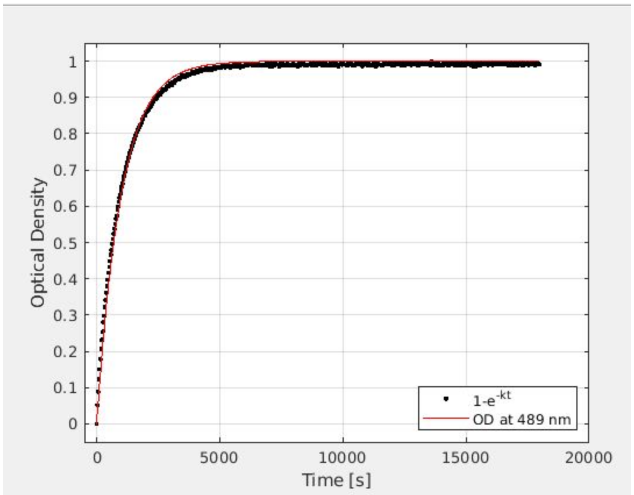

Figure S2. Kinetic trace of the absorption at 489nm measuring the thermal recovery rate constant for the Trans-to-Cis conversion at 298 K and pH 8.4, yielding a value of 1.0x10^{-3} s^{-1}.

This means the minimum expected cis population before X-ray probing of the crystal is calculated to be 12.5%.

Because the calculated temperature jump is sensitive to the value used for the concentration of the trans state, we consider the possibility of experimental and fitting uncertainty of the laser profile and power density. This is estimated by assuming a difference in profile that could be the conversion of FWHM to the 1/e ^{2} profile. This would give an estimate of 0.22 W/cm^2 as a possible lower bound value for the power density. Consequently, a conversion rate constant of 2.2 s^{-1} results in the

calculation of a minimum of 16.6% Cis population in addition to 4.3% thermal conversion, which equals 20.9%. Taking this value as an upper limit by assuming errors in the power density determination, the temperature jump calculations are done assuming a value of 12.5 +/- 8.4 % for the Cis population. This uncertainty is propagated for the calculation of the temperature jump, section S4.3 below.

S4. MODELING OF PHOTOCONVERSION IN CRYSTALS

S4.1. Single Flash Yield and Error Propagation

The population change (*N*) due to the single 405 nm flash was calculated from the photodiode signal using the Beer-Lambert law:

$$N = \frac{[\mathrm{Cis}]}{[\mathrm{CisTotal}]} = \frac{\log_{10}(I_0 = I_{[\mathrm{Cis}]})}{\log_{10}(I_0 = I_{[\mathrm{CisTotal}]})}$$

Where [Cis] is the concentration of the cis conformer and [CisTotal] the fully converted concentration (*i.e.* total sample concentration), I_0 is the initial intensity (or full trans), $I_{[Cis]}$ is the population due to the 405 nm flash and $I_{[CisTotal]}$ is the fully converted signal level of the cis state, as shown in figure S3. The standard error in the conversion, *N* was calculated as:

$$\delta N = \sqrt{\frac{\left(I_{[\mathrm{CisTotal}]}I_{[\mathrm{Cis}]}\delta I_0 \log_{10}\left(\frac{I_{[\mathrm{Cis}]}}{I_{[\mathrm{CisTotal}]}}\right)\right)^2 + \left(\delta I_{[\mathrm{Cis}]}I_0 \log_{10}\left(\frac{I_0}{I_{[\mathrm{CisTotal}]}}\right)\right)^2 + \left(\delta I_{[\mathrm{CisTotal}]}I_{[\mathrm{Cis}]}I_0 \log_{10}\left(\frac{I_0}{I_{[\mathrm{Cis}]}}\right)\right)^2}{\left(I_{[\mathrm{CisTotal}]}I_{[\mathrm{Cis}]}I_0 \log_{10}\left(\frac{I_0}{I_{[\mathrm{Cis}]}}\right)^2\right)^2}}$$

Where $\delta I_{[\mathrm{CisTotal}]}$, $\delta I_{[\mathrm{Cis}]}$ and δI_0 are the standard error in the respective values, calculated as: $\delta X = \frac{\sigma(X)}{\sqrt{n}}$ where $\sigma(X)$ is the standard deviation in value *X* over *n* observations.

The energy density in mJ/mm^2 was calculated as:

$$E = E_{\max} \times 10^{-ND}$$

Where E_{max} is the energy density with $ND = 0$ where *ND* is the optical density of the neutral density filter being used. The standard error in energy density, *E* can be calculated as:

$$E = \sqrt{10^{-2ND}(E^2 \delta ND^2 + \delta E^2)}$$

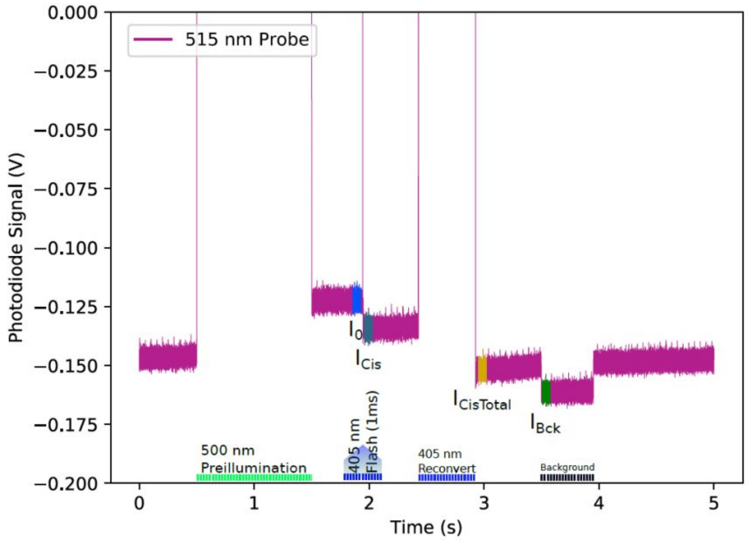

Figure S3: Plot of a typical flash photolysis trace. Showing the illumination scheme (bottom) and which points in the trace correspond to the values used to calculation the single flash yield. I_{Bck} was the no light background value subtracted from all point to correct for the offset on the trace.

S4.2. Flash Photolysis calculation

To fit the flash-photolysis data a model was derived based on first order reaction kinetics. On millisecond times scales the cis-trans isomerization can be considered a single-step process:

$$\text{Trans} \xrightarrow{k_{\text{switch}},\, k_{\text{TH}}} \text{Cis}$$

The switching rate constant depends on the photon flux q, the proportion of absorbed photons χ in a sample of absorbance, A, the quantum yield of Trans/Cis switching,Φ, and the thermal rate, k_{TH}:

$$-\frac{d[Trans]}{dt} = \frac{q\,\chi\phi}{V} + k_{TH}[Trans]$$

For the case where OD « 1 a Taylor expansion can be used to approximate the absorbed fraction as:

$$1 - 10^{-A} \approx A \ln(10)$$

And

$$\chi \cong \ln(10)\epsilon[Trans]l$$

Where ε is the molar extinction coefficient (M^{-1} cm^{-1}) and l the path length (cm)

Using this approximation and the Beer-Lambert law, the switching rate can be rewritten as:

$$-\frac{d[Trans]}{dt} = \left(\frac{\ln(10)}{10}\right)E\phi\epsilon[Trans] + k_{TH}[Trans] = k_1[Trans] + k_{TH}[Trans]$$

Where k_1 is a first order rate constant

The rate of thermal recovery of the trans state during the flash-photolysis measurement is much smaller than the rate of photo-induced switching, so can be neglected in the 2ms flash duration.

For a Fluence (mJ mm^{-2}) delivered in the 2ms flash to drive the Trans-to-cis reaction

$$ln\left(\frac{[Trans]_0}{[Trans]_F}\right) = k_1 F$$

Where k is a fluence-based first-order rate constant in the units of mm^2/mJ[8].

$$k = \frac{\ln(10)\phi\varepsilon}{10}$$

The data were fitted (Figure S4) using the orthogonal distance regression package in scipy.[57] This minimizes residuals by accounting for errors in both the dependent and independent variables when fitting, which was appropriate due to the significant errors in both the energy density and the measured intensity.

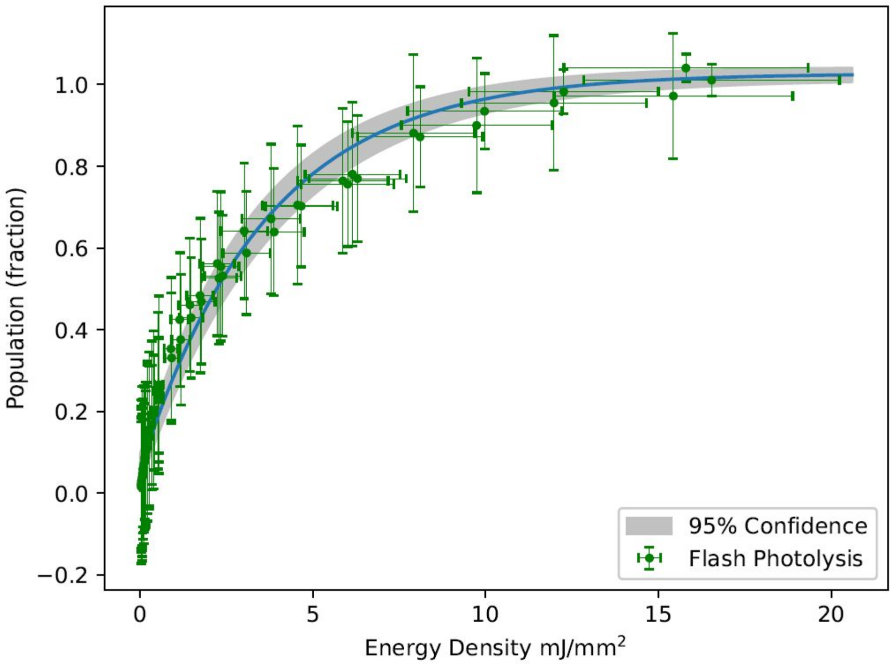

Figure S4: Population change plotted against energy density obtained from optical flash photolysis data collection.

S4.3. X-Ray Crystallography Photolysis Modelling

The crystallography rate measurements can be considered a three step process:

$$\text{Cis} + \text{Trans} \xrightarrow{\text{Preconvert}} \text{Cis} + \text{Trans} \xrightarrow{\text{Flash}} \text{Cis} + \text{Trans} \xrightarrow{\text{X}-\text{ray Probe}} \text{Cis} + \text{Trans}$$

As mentioned in the section S3 above, the initial preconversion step and thermal recovery before measurement should lead to an initial cis population of 12.5% which was constant through the experiment.

The temperature jump ΔT is calculated from the Arrhenius equation, using E_a=91 +/- 5 KJ mol^{-1} and k_1=4.08 x 10^{-4} s^{-1} at a temperature of 293 K.

$$\Delta T = T_2 - 293K = {-1} \Big/ \left(\frac{R ln\left(\frac{k_2}{k_1}\right)}{E_a} - \frac{1}{293} \right) - 293$$

Where k_2 is calculated from the concentration [Cis] measured at 0 mJ/mm^2 power density (Figure 4) that provides the converted fraction X

$$x = \frac{[Cis] - 0.125}{0.875}$$

And

$$k_2 = \frac{(1 - e^{-x})}{0.01}$$

For the 0.01 s time point that measures [Cis].

The RMS error for the Ea dominates the error on the calculation of ΔT, more strongly than the errors in the determination of the cis concentration from spectroscopy (Section S3) and the uncertainly of the crystallographic quantification. The uncertainty of +/- 0.05 fraction of the [Cis] determined from the crystallographic methods at 0mJ/mm^2 adds to the uncertainty typically between 2 and 5 degrees K. The resulting average and error is reported in Table 1 in the main manuscript.

To illustrate, the uncertainties in the values for Ea, the Cis concentration at zero power density for the 405nm laser from spectroscopy, and the uncertainty in the crystallographic quantification methods propagate as follows, as shown in tables S1,S2 and S3 below.

Method	[Cis]-crystallographic	[Cis]-spectroscopy	ΔT (for Ea=91.5 KJ/mol)	ΔT (for Ea=81.5 KJ/mol)	ΔT (for Ea=101.5 KJ/mol)
R_{free}	0.56	0.125	128.8 K	111.3 K	152.9 K
$F_{extrapolated}$	0.33	0.125	118.9 K	103.0 K	140.5 K
F_{work}	0.25	0.125	112.1 K	97.4 K	132.0 K
Fo-Fc	0.29	0.125	115.9 K	100.6 K	136.8 K

Table S1. Calculated temperature jump values for the mean value of 0.125 determined for the Cis concentration from spectroscopy, for the mean (91.5 KJ/mol), minimum (81.5 KJ/mol) and maximum (101.5 KJ/mol) values for the activation energy Ea.

Method	[Cis]-crystallographic	[Cis]-spectroscopy	ΔT (for Ea=91.5 KJ/mol)	ΔT (for Ea=81.5 KJ/mol)	ΔT (for Ea=101.5 KJ/mol)
R_{free}	0.56	0.041	129.9 K	112.2 K	154.2 K

$F_{extrapolated}$	0.33	0.041	122.3 K	105.9 K	144.8 K
F_{work}	0.25	0.041	117.9 K	102.2 K	139.3 K
Fo-Fc	0.29	0.041	120.3 K	104.2 K	142.2 K

Table S2. Calculated temperature jump values for the minimal estimated value of 0.041 determined for the Cis concentration from spectroscopy, for the mean (91.5 KJ/mol), minimum (81.5 KJ/mol) and maximum (101.5 KJ/mol) values for the activation energy Ea.

Method	[Cis]-crystallographic	[Cis]-spectroscopy	ΔT (for Ea=91.5 KJ/mol)	ΔT (for Ea=81.5 KJ/mol)	ΔT (for Ea=101.5 KJ/mol)
R_{free}	0.56	0.209	127.4 K	110.1 K	151.1 K
$F_{extrapolated}$	0.33	0.209	113.0 K	98.2 K	133.2 K
F_{work}	0.25	0.209	98.1 K	85.6 K	114.9 K
Fo-Fc	0.29	0.209	107.5	93.5 K	126.3 K

Table S3. Calculated temperature jump values for the maximal estimated value of 0.209 determined for the Cis concentration from spectroscopy, for the mean (91.5 KJ/mol), minimum (81.5 KJ/mol) and maximum (101.5 KJ/mol) values for the activation energy Ea.

S5. KNIFE EDGE LASER FOCAL SPOT SIZE DETERMINATION

To ensure accurate determination of power density of the 405nm laser, both in the home lab and at the synchrotron beamline, knife edge scans were performed to ascertain the spot sizes. Traces were fitted as described in[9] using SciPy in python to extract the FWHM of the focus profile.

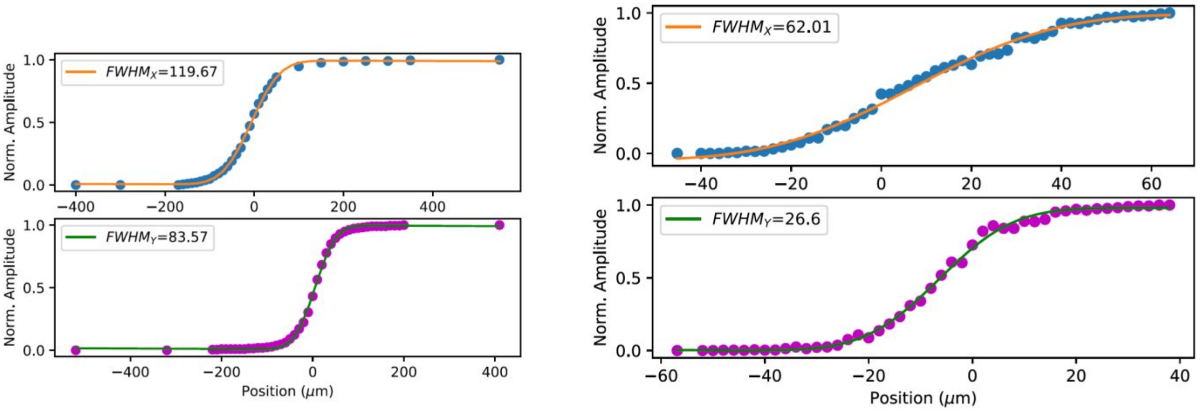

Figure S5: Knife edge scans of the laser focus at the home lab (left) and P14.2 (T-REXX) at PETRA III (right).

S6. SSX ENERGY TITRATION CHROMOPHORE-OMIT MAPS

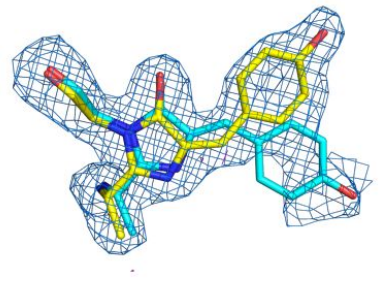

(a) 0 mJ/mm^2

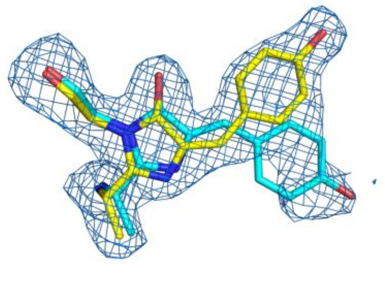

(b) 0.1 mJ/mm^2

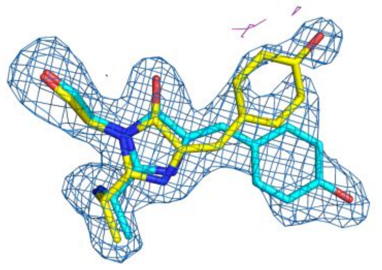

(c) 0.53 mJ/mm^2

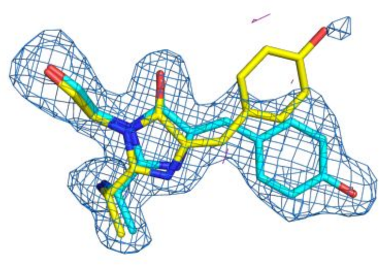

(d) 1.78 mJ/mm^2

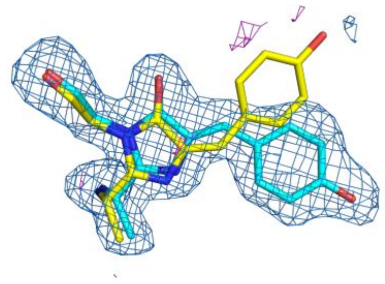

(e) 6.74 mJ/mm^2

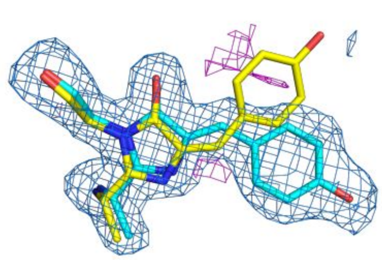

(f) 14.43 mJ/mm^2

Figure S6: $2F_{obs}$-F_{calc} chromophore-omit maps of varying laser powers plotted at a sigma level of ± 3, calculated by phasing a 50:50 model of the cis and trans structures with the chromophore occupancy set to 0. Shown in blue is the positive density and in magenta the negative density.

S7. SSX ENERGY TITRATION OCCUPANCY DETERMINATION BY MINIMISATION OF R_{FREE} AND R_{WORK}.

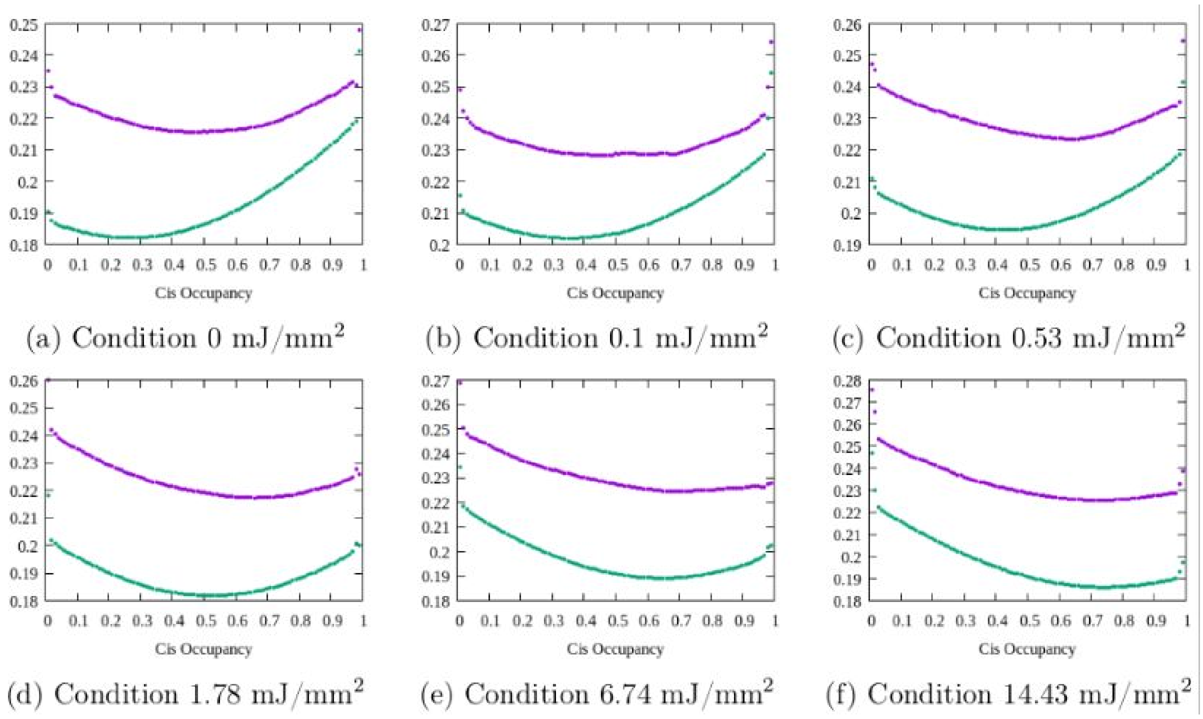

(a) Condition 0 mJ/mm^2 (b) Condition 0.1 mJ/mm^2 (c) Condition 0.53 mJ/mm^2

(d) Condition 1.78 mJ/mm^2 (e) Condition 6.74 mJ/mm^2 (f) Condition 14.43 mJ/mm^2

Figure S7: Plots of R_{Free} (purple) and R_{Work} (green) against the cis (1-trans) occupancy level for all illumination powers.

S8. SSX ENERGY TITRATION OCCUPANCY DETERMINATION USING EXTRAPOLATED STRUCTURE FACTORS

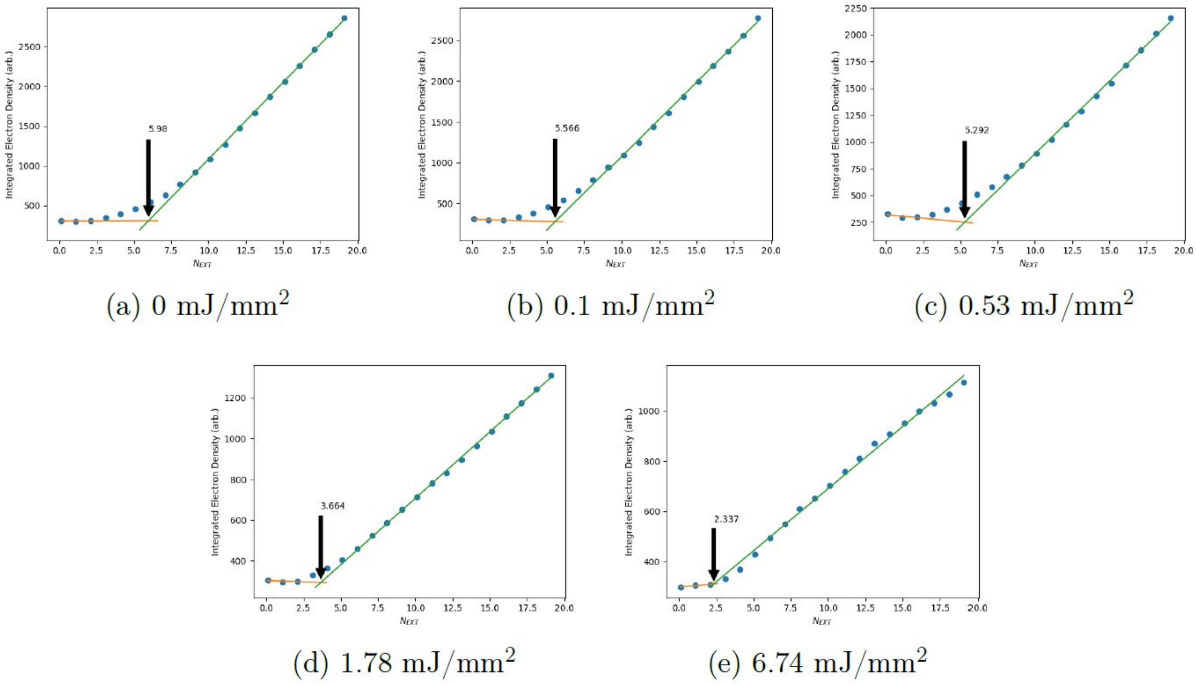

(a) 0 mJ/mm^2 (b) 0.1 mJ/mm^2 (c) 0.53 mJ/mm^2

(d) 1.78 mJ/mm^2 (e) 6.74 mJ/mm^2

Figure S8: Plots of integrated electron density in arbitrary units against multiples of added difference electron density N_{EXT} for different illumination powers. The intersection of the two linear regions corresponds to the characteristic value of N_{EXT} related to PT = 200/N_{EXT}.

S9. CRYSTALLOGRAPHIC DATA TABLES

Table S4: TR-SSX diffraction data statistics collected for all pump energy densities.

Energy Density (mJ/mm^2)	0	0.10	0.53	1.78	6.74	14.43
PDB code:	8UL0	8UL1	8UL2	8UL3	8UL4	8UL5
Light source:	PETRA III	PETRA III	PETRA III	PETRA III	PETRA III	PETRA III
Beamline:	EH14.2	EH14.2	EH14.2	EH14.2	EH14.2	EH14.2
Space Group	$P2_12_12_1$	$P2_12_12_1$	$P2_12_12_1$	$P2_12_12_1$	$P2_12_12_1$	$P2_12_12_1$
Cell Dimensions,						
a,b,c (Å):	39.6, 74.5, 78.9	39.6, 74.5, 78.9	39.78, 74.98, 79.61	39.77, 75.01, 79.59	39.84, 74.96, 79.52	39.82, 75.02, 79.56
α, β, γ (°)	90, 90, 90	90, 90, 90	90, 90, 90	90, 90, 90	90, 90, 90	90, 90, 90
Indexed Patterns:	12649	10555	14166	18204	13585	10817
Resolution Limits (Å):	78.69-1.70 (1.76-1.70)	78.69-1.70 (1.76-1.70)	78.69-1.70 (1.76-1.70)	78.69-1.70 (1.76-1.70)	78.69-1.70 (1.76-1.70)	78.69-1.70 (1.76-1.70)
Uniq. Refl. Indices:	26098	26098	26098	26098	26098	26098
Merged Reflections:	1632100 (14441)	1222429 (15103)	1312430 (11164)	2281927 (18313)	2335166 (34153)	1532230 (14220)
Completeness (%):	99.38 (94.27)	99.39 (95.32)	98.98 (91.47)	99.76 (97.74)	99.94 (99.65)	99.32 (94.04)
Signal to noise:	3.884 (0.86)	3.114 (0.48)	3.586 (0.72)	4.552 (0.88)	4.099 (0.55)	3.608 (0.97)
Wilson B factor ($Å^2$):	23.89	28.14	26.45	23.39	25.64	24.35
R_{Split} (%):	17.81 (185.33)	21.85 (351.74)	19.10 (341.10)	15.45 (209.70)	17.00 (269.17)	19.25 (235.94)
CC*:	0.99 (0.46)	0.99 (0.28)	0.99 (0.08)	0.99 (0.50)	0.99 (0.39)	0.99 (0.42)
$CC_{1/2}$:	0.97 (0.12)	0.96 (0.04)	0.97 (0.00)	0.98 (0.14)	0.98 (0.08)	0.97 (0.09)
Fourier Shell Coefficient (FSC)	(0.632)	(0.55)	(0.53)	(0.49)	(0.56)	(0.60)
**Refinement:**						
No. reflect. all/free	24164 / 1215	23910 / 1141	23983 / 1151	24180 / 1158	24154 / 1156	24122 / 1152
R/R-free:	0.171 / 0.211	0.176 / 0.219	0.170 / 0.216	0.156 / 0.195	0.170 / 0.209	0.178 / 0.214
Bonds (RMS):	0.0097	0.0087	0.0097	0.0113	0.01	0.0099
Angles (RMS):	1.58	1.554	1.611	1.669	1.632	1.642

Note: numbers in parentheses refer to the highest resolution shell

S10. SUPPORTING INFORMATION REFERENCES

(1) Bury, C. S.; Brooks-Bartlett, J. C.; Walsh, S. P.; Garman, E. F. Estimate Your Dose: RADDOSE-3D. *Protein Science* **2018**, *27* (1), 217–228. https://doi.org/10.1002/pro.3302.

(2) Zeldin, O. B.; Gerstel, M.; Garman, E. F. RADDOSE-3D: Time- and Space-Resolved Modelling of Dose in Macromolecular Crystallography. *J Appl Crystallogr* **2013**, *46* (4), 1225–1230. https://doi.org/10.1107/S0021889813011461.

(3) Warren, A. J.; Axford, D.; Owen, R. L. Direct Measurement of X-Ray-Induced Heating of Microcrystals. *J Synchrotron Radiat* **2019**, *26* (4), 991–997. https://doi.org/10.1107/S1600577519003849.

(4) Kriminski, S.; Kazmierczak, M.; Thorne, R. E. Heat Transfer from Protein Crystals: Implications for Flash-Cooling and X-Ray Beam Heating. *Acta Crystallogr D Biol Crystallogr* **2003**, *59* (4), 697–708. https://doi.org/10.1107/S0907444903002713.

(5) Miyazaki, Y.; Matsuo, T.; Suga, H. Low-Temperature Heat Capacity and Glassy Behavior of Lysozyme Crystal. *Journal of Physical Chemistry B* **2000**, *104* (33), 8044–8052. https://doi.org/10.1021/jp0007686.

(6) Fujiwara, S.; Maki, S.; Tanaka, S.; Maekawa, R.; Masuda, T.; Hagiwara, M. Measurements of Thermal Conductivity and Thermal Diffusivity of Hen Egg-White Lysozyme Crystals Using a Short Hot Wire Method. In *AIP Conference Proceedings*; 2017; Vol. 1865, p 40009. https://doi.org/10.1063/1.4993351.

(7) Hutchison, C. D. M.; Baxter, J. M.; Fitzpatrick, A.; Dorlhiac, G.; Fadini, A.; Perrett, S.; Maghlaoui, K.; Lefèvre, S. B.; Cordon-Preciado, V.; Ferreira, J. L.; Chukhutsina, V. U.; Garratt, D.; Barnard, J.; Galinis, G.; Glencross, F.; Morgan, R. M.; Stockton, S.; Taylor, B.; Yuan, L.; Romei, M. G.; Lin, C.-Y.; Marangos, J. P.; Schmidt, M.; Chatrchyan, V.; Buckup, T.; Morozov, D.; Park, J.; Park, S.; Eom, I.; Kim, M.; Jang, D.; Choi, H.; Hyun, H.; Park, G.; Nango, E.; Tanaka, R.; Owada, S.; Tono, K.; DePonte, D. P.; Carbajo, S.; Seaberg, M.; Aquila, A.; Boutet, S.; Barty, A.; Iwata, S.; Boxer, S. G.; Groenhof, G.; van Thor, J. J. Optical Control of Ultrafast Structural Dynamics in a Fluorescent Protein. *Nat Chem* **2023**, *15* (11), 1607–1615. https://doi.org/10.1038/s41557-023-01275-1.

(8) Bolton, J. R.; Mayor-Smith, I.; Linden, K. G. Rethinking the Concepts of Fluence (UV Dose) and Fluence Rate: The Importance of Photon-Based Units - A Systemic Review. *PhotochemPhotobiol* **2015**, *91* (6), 1252–1262. https://doi.org/10.1111/php.12512.

(9) de Araújo, M. A.; Silva, R.; de Lima, E.; Pereira, D. P.; de Oliveira, P. C. Measurement of Gaussian Laser Beam Radius Using the Knife-Edge Technique: Improvement on Data Analysis. *Appl Opt* **2009**, *48* (2), 393. https://doi.org/10.1364/AO.48.000393.
